# Supplementary material for: Augmented reality-based affective training for improving care communication skill and empathy
Source: PLoS One. 2023 Jul 10;18(7):e0288175. doi: 10.1371/journal.pone.0288175 (PMC10332577; doi:10.1371/journal.pone.0288175)
Supplement: Attachment — Submitted filename: Response to reviewers.pdf [file pone.0288175.s001.pdf]

We thank all the reviewers for their careful consideration and insightful comments. We have updated our manuscript in accordance with these comments, as explained below. In the main text, updated portions are indicated in red.

Reviewer #1:

1. Research questions, that drive the paper, should be built in the introduction from an ongoing and pertinent bibliography (up to 2022-23) and these should be of global interest and not focused on a particular local problem. Identifying a research gap is the most important by indicating in-text some newer references that are significant to your particular field of research.
2. The problem statement needs to be clear into Introduction. Does the relevant literature refer the potentials of using "conventional" instead of typical AR-supported activities to drive us there?
3. Some indicative literature review articles regarding the use of AR-supported learning that the same authors read maybe of great importance to readers in order to identify the research "gap" more easily without mention too many previous studies.
4. The authors need to provide any potentials regarding the use of AR settings. Are there any literature reviews to give us a point of view about this investigation? In other words, the authors need to "combine" any key word that investigate with any relevant study that can be integrated in-text as "background".

In the Introduction section, we have added references to several of the latest studies regarding conventional affective communication intervention, such as [6], [11], and [12]. In addition, the problem statement (research question) paragraph has been added in the Introduction as well. Furthermore, we introduced the latest survey papers regarding AR-based skill training such as [13]-[16], which include several application scenarios using AR for training.

5. Any information respecting participants' background can be beneficial for readers to understand better the level of technological literacy can overcome this "novelty effect" using AR.

We have added a description stating the participants had average computing literacy but no experience using VR/AR.

6. The authors should make explicit suggestions about how their study affects the design or use of AR systems. Is there something new about a particular theory, or is there evidence of theory advancement?

In the revised version of our paper, we have introduced the concept and detailed flowchart of how the agent responds in section 2. We believe this can serve as a design reference for the development of future systems.

7. The added value of authors' work is not clear in terms of proper and current research (up to 2022-23), because several references are not up-to-date.
8. Authors should answer the main research question in the conclusions and discussion. Please provide a reasonable need to read your work's results than previous ones or simply answer what we learned compared with current, significant research (up to 2022 should be your work's "significance").

In the updated version, we have introduced the latest related studies (including surveys) and described the position of our paper in the Introduction and Discussion sections.

9. How general are your results and how do you believe that such findings have to be of global interest? Please relate these with your limitations and Discussion that is not exist. Why?
10. Are there any points of view related to the consequences of this study's limitations that may have an impact on their findings?
11. Conclusions and limits are too short for such a study.
12. Practical and educational implications are not provided.

We have added a section regarding the limitation of the number of participants and duration of the intervention. However, as we have written, our work has a relatively large number of participants in this sort of study using AR. Regarding the potential of our work, we have added the position of the paper in the Discussion section.

Reviewer #2:
This is a well written paper that presents a relevant description of a new methodology. The authors useful information has been provided. this papare may

be of great interest to Plos One readers and all that are interested in nwe methods of teching and learning.

Thank you very much for kindly understanding the potential of the paper.

Reviewer #3:

[2] I would like to see a well-developed discussion (minimum two pages) comparing and contrasting solution/results presented in the work with existing work and then a subsection of it presenting contributions to theory/knowledge/literature (at least one to two paragraphs) and followed by a subsection on Implications for practice (at least one page). In these paragraphs authors should compare their research approach with previous research, citing references of others' research.

[4] The authors must add a new section called Background Literature which establishes the context of the research. This section explains why this particular research topic is important and essential to understanding the main aspects of the study. Usually, the background forms the first section of a research article/thesis and justifies the need for conducting the study and summarizes what the study aims to achieve.

As other reviewers have provided similar comments, we have added the introduction, discussion, and limitation sections to meet your request, though we did not follow the exact format suggested. We introduced more recent related studies and described the position of our paper as well as the limitations.

[3] The overall document should be checked for grammar, syntax and typos errors. Based on the above comments, I strongly believe that the authors will improve the quality of their manuscript given that they will make a detailed revision of the manuscript based on the provided comments

Thank you for the advice. We will carefully proofread and have the manuscript checked by external proofreaders.

[5] The authors must add a pseudocode diagram in section 2 explaining how the software algorithm is implemented. What software is used to implement the VR platform?

We have added the flowchart of the avatar's motion in section 2. We used a Unity 2020 environment for the development.

[6] In the Section 2, the authors should briefly highlight the main developments of their research topic and identify the main gaps that need to be addressed. In other words, this section should give an overview of your study. The section should be organized as: What is known about the broad topic?, What are the gaps or missing links that need to be addressed?, What is the significance of addressing those gaps?, and What are the rationale and hypothesis of your study?. Please rewrite this section.

We have described our aim and hypotheses at the end of the first section (Introduction), specifically in lines 12-43, page 2. We also introduced existing literature regarding affective communication training and outcomes and VR/AR systems. Lastly, we described our research questions and hypotheses in lines 53-73.
